# The Role of Liver-Directed Therapy in Metastatic Colorectal Cancer

**DOI:** 10.1007/s11888-018-0409-6

**Published:** 2018-09-13

**Authors:** Hon Lyn Tan, Matilda Lee, Balamurugan A. Vellayappan, Wee Thong Neo, Wei Peng Yong

**Affiliations:** 1 Department of Haematology-Oncology, National University Cancer Institute, Singapore (NCIS), National University Health System, 1E Kent Ridge Road, Singapore, 119228 Singapore; 2 Department of Radiation Oncology, National University Cancer Institute, Singapore (NCIS), National University Health System, 1E Kent Ridge Road, Singapore, 119228 Singapore; 3 Department of Diagnostic Imaging, National University Health System, 1E Kent Ridge Road, Singapore, 119228 Singapore

**Keywords:** Colorectal cancer liver metastases, Colorectal cancer, Liver metastases, Liver-directed therapy

## Abstract

**Purpose of Review:**

Colorectal cancer liver metastasis is a major clinical problem, and surgical resection is the only potentially curative treatment. We seek to discuss various liver-directed therapy modalities and explore their roles in the evolving realm of treatment strategies for metastatic colorectal cancer.

**Recent Findings:**

Clinical outcomes for patients with colorectal cancer liver metastases have improved as more patients undergo potentially curative resection and as the armamentarium of systemic treatment and liver-directed therapies continues to expand. Liver-directed therapies have been developed as adjuncts to improve resectability, employed in the adjuvant setting to potentially reduce local recurrence rates, and utilized in the palliative setting with the aim to improve overall survival.

**Summary:**

Ongoing research is expected to validate the role of these evolving therapeutic options, and determine how best to sequence and when to apply these therapies.

## Introduction

The most common site of colorectal cancer metastases is the liver. Liver metastases are present in 20–35% of patients at diagnosis and in up to 70% of patients at death [1]. Uncontrolled liver metastases account for approximately 50% of colorectal cancer death [2]. Systemic therapy alone is, in general, non-curative and does not produce a sustained response. Surgical resection is recommended for isolated liver metastases, with the goals of improving relapse-free survival, and of potential cure [3]. Earlier studies showed a 30% 5-year survival rate in patients who underwent hepatic resection for one to three liver metastases. [4]

Factors that determine resectability include lesion size, number, location, and hepatic reserve. While surgical techniques have improved to include patients with higher burden of hepatic disease, several non-surgical liver-directed therapies have now become possible. These therapies are utilized in patients with isolated liver metastases either as treatment for unresectable disease, to convert to resectable disease, or to reduce the risk of recurrence after surgery.

We aim to discuss various liver-directed therapy modalities, including hepatic arterial infusion (HAI) therapy, radiation therapy including stereotactic body radiotherapy (SBRT) and selective internal radiation therapy (SIRT), transarterial chemoembolization (TACE), and radiofrequency ablation (RFA) in detail, and explore their roles in evolving realm of treatment strategies for metastatic colorectal cancer.

## Hepatic Artery Infusion Chemotherapy

In hepatic artery infusion (HAI), chemotherapy is administered into the hepatic artery by a surgically implanted pump/port or a percutaneously placed catheter. This technique relies on the differential blood supply to the liver, where liver metastases derive more than 80% of their blood supply from the hepatic artery circulation, while normal hepatocytes derive theirs from the portal circulation. An ideal drug would have a short half-life and high first-pass metabolism allowing for increased drug exposure within liver tumors while keeping the systemic drug concentration low.

Floxuridine (FUDR) is the most widely used agent in the USA because of its favorable pharmacokinetics. Up to 99% of FUDR is extracted by the liver during the first pass which results in a hepatic/systemic ratio of 100–400, compared to a hepatic extraction rate of 19–55% for 5-fluorouracil (5-FU) resulting in a hepatic/systemic ratio around 10 [5].

### The Role of HAI in Unresectable Liver Disease

Several early reports demonstrated improved response rates with HAI FUDR or HAI 5-FU alone over systemic chemotherapy in patients with unresectable liver metastases with three meta-analyses publishing conflicting reports. The largest meta-analysis, comprising 1277 patients in 10 randomized studies, reported a notably higher response rate with HAI compared to systemic chemotherapy (42.9% vs 18.4%). There was a trend towards improved survival, but that did not reach statistical significance (15.9 months vs 12.4 months, HR 0.90, *p* = 0.24) [6–8]. The best available systemic chemotherapeutic agent then was 5-FU, which was the comparator in most of these studies.

Newer agents have now become available for HAI, such as irinotecan or oxaliplatin alone or in combination. A phase I study, involving 31 patients, used HAI irinotecan and oxaliplatin with bolus FUDR given every 4–8 weeks. This achieved a response rate of 61% and median survival of 24.8 months. These patients also received IV FUDR with leucovorin on days 1–3 after the HAI procedure [9]. French investigators studied combination HAI oxaliplatin, 5-FU, and irinotecan with intravenous cetuximab in 64 previous patients with unresectable liver metastases from *RAS* wild-type colorectal cancer in a phase II study. Response rate was 40.6% and 29.7% patients underwent R0-R1 resection. Median survival was 25.5 months [10].

### The Role of HAI in Combination With Systemic Chemotherapy

While there are no published phase III trials examining systemic chemotherapy in combination with HAI, several phase I/II trials have evaluated the safety and efficacy of combined therapy. Results suggest improved response rates than either treatment alone. Combined therapy may also reduce the risk of extrahepatic progression. A study using HAI FUDR plus systemic irinotecan in previously treated patients produced a response rate of 74% and a median survival of 20 months [11]. A phase I study from MSKCC using concurrent HAI FUDR with dexamethasone plus systemic oxaliplatin with irinotecan or 5-FU/LV (FOLFOX) demonstrated further improvement. Of those who received HAI FUDR plus IV oxaliplatin and irinotecan, 19 out of 21 (90%) had a partial response and median survival was 35.8 months [12]. A Chinese study, in which half of the 35 patients enrolled had extrahepatic disease, studied HAI FUDR plus IV mFOLFOX6, and produced a response rate of 68.6% and a median survival of 25 months [13].

### The Role of HAI in the Neoadjuvant Setting

The role of HAI as conversion therapy deserves further evaluation. A MSKCC study had 49 patients with unresectable liver metastases treated with HAI FUDR plus systemic oxaliplatin and irinotecan. A response rate of 92% was observed, with 47% of patients subsequently undergoing liver resection with curative intent. Median overall survival (OS) for previously treated and treatment-naive patients was 35 and 50.8 months respectively [14]. More recently, a phase II prospective study evaluating 64 patients treated with HAI and systemic chemotherapy reported its updated analysis showing a 52% conversion rate and an OS of 38 months at a median follow-up of 81 months [15•].

### The Role of HAI After Resection of Hepatic Metastasis

In 1999, a US multicenter study showed a survival benefit of adjuvant systemic 5-FU with or without LV plus HAI over systemic chemotherapy alone after resection [16]. The median survival was 72.2 months in the combined treatment group vs 59.3 months in the systemic chemotherapy alone group and 2-year liver recurrence free survival was 90% and 60% (*p* = < 0.001) respectively. However, extrahepatic recurrence rates were similar in both groups. An intergroup trial evaluating adjuvant HAI FUDR alternating with systemic capecitabine and oxaliplatin after resection of colorectal liver metastases showed promising results, meeting the pre-specified endpoint of > 85% survival at 2 years [17]. However, the phase III trial that followed comparing capecitabine and oxaliplatin with or without HAI FUDR closed early due to poor accrual.

Adjuvant HAI in combination with contemporary chemotherapy such as irinotecan [18] and oxaliplatin [19] have been investigated in phase I/II studies, but large randomized controlled trials are lacking. The addition of bevacizumab was also studied; however, no improvement in RFS was demonstrated at the expense of increased biliary toxicity [20].

Drug-related toxicities of HAI FUDR include biliary sclerosis (2–5.5%) [21] and gastric ulceration requiring close monitoring of liver function and the use of routine proton-pump inhibition. Technical complications of the procedure such as arterial thrombosis, catheter occlusion, or dislodgement may occur. While the NCCN guidelines indicate that HAI with or without systemic 5-FU and leucovorin is an option after liver resection at centers with experience in both the surgical and oncological aspects of this treatment [22], two significant limitations are the requirement of specific expertise and that FUDR is not readily available in many countries.

The optimal combination of therapeutic agents used with HAI and its place among the various options available to achieve the best treatment outcomes deserve to be further studied and clarified.

## Stereotactic Body Radiation Therapy

Traditionally, the role of radiation therapy in liver metastases has been for palliation of pain. The tolerance of the whole liver to radiation is limited the dose to 30 Gy [23], which is unable to produce sustained local control. Technological advances over the years, with improvements in target localization, patient immobilization, motion management, and delivery of conformal radiation, have allowed the use of high doses of radiation to ablate and control liver metastases. Moreover, mounting evidence show that high doses of radiation can be delivered to a small volume of liver without causing toxicity [23]. SBRT, typically using doses ranging from 45 to 60 Gy over three to five fractions (given over 1–2 weeks), is delivered conformally to the target while sparing normal liver parenchyma.

The use of SBRT for liver metastases has been extrapolated from the long history of radiosurgery used in intracranial tumors. However, in contrast to intracranial tumors, liver metastases are subject to significant organ motion, at times exceeding 2 cm. Techniques to account for, and reduce organ motion, have been essential for the safe delivery of SBRT. These include 4DCT scans, image-guidance with onboard cone-beam CT (with or without the use of implanted fiducials), abdominal compression devices, voluntary breath-holds and delivery systems which track organ motion such as Cyberknife, or gate radiation delivery during certain phases of the respiratory cycle. A comprehensive review of the above techniques is beyond the scope of this review, and interested readers are recommended to read further [24].

Being a non-invasive treatment, with a convenient schedule, SBRT is an attractive proposition, for it does not delay systemic therapy unduly. Patient selection is crucial, and factors which determine suitability of SBRT include performance status, volume of hepatic disease, distance of target from critical structures (such as bowel or biliary tract), and amount of hepatic reserve (preferably with a liver volume > 1000 cc, with an aim to spare at least 700 cc from doses exceeding 15 Gy) [23]. Although there is no size limit for SBRT, most studies have limited SBRT to three liver metastases or fewer, with maximal size up to 6 cm [25].

At present, there is no level 1 evidence for SBRT demonstrating improved survival. However, numerous phase I–II prospective studies suggest a 1-year local control of 70 to 100% at 1 year, and 60 to 90% at 2 years [26–29]. The high biologically equivalent doses, and high dose per fraction, afford sustained local control and may have novel ways of cell-killing such as immune activation [30] and endothelial apoptosis [31]. Besides radiation dose, factors which influence local control include tumor volume and mutation status [29] (such as K-RAS and TP53) and prior systemic therapy. SBRT is generally well tolerated, with severe toxicity well below 5% [27, 28]. In summary, patients who are unsuitable for liver resection should be considered for treatment with focal ablative therapies such as SBRT, with goals of improving local control and survival.

## Selective Internal Radiation Therapy

SIRT delivers targeted radiation via the hepatic artery using radioactive isotope-tagged resin or glass microspheres. The yttrium-90 microspheres lodge in hepatic arterioles that feed liver metastases. Maximum tissue penetration for the beta-emitter yttrium-90 is 11 mm, sparing most normal liver parenchyma.

### The Role of SIRT in the First-Line Setting

In the SIRFLOX study, patients with unresectable liver-confined metastases were randomized to receive SIRT in combination with modified FOLFOX chemotherapy (with or without bevacizumab) or chemotherapy alone. Overall PFS was similar in both treatment arms (10.7 vs 10.2 months; *p* = 0.428) although liver-specific PFS was significantly prolonged with the addition of SIRT (20.5 in the SIRT arm vs 12.6 months in the control arm; *p* = 0.002) [32•]. Combined analysis of three parallel studies, i.e., SIRFLOX, FOXFIRE, and FOXFIRE-Global, included 1103 patients. At median follow-up of 43 months, there was no demonstrable difference in median OS (22.6 vs 23.3 months, HR 1.04, 95% CI 0.9–1.1) or PFS (11 versus 10.3 months, HR 0.90, 95% CI 0.79–1.02) despite higher response rates and improved liver-specific PFS. Combination treatment was associated with more grade 3/4 adverse events, in particular hematologic toxicity. Of the 11 treatment related deaths, 8 were in the combination treatment group with 3 attributed to radiation induced liver disease [33••]. Overall survival in patients with right-sided primary tumors treated with FOLFOX and SIRT was seen to be longer compared to chemotherapy alone.

In the second-line setting, the ongoing phase III EPOCH study aims to address the efficacy and safety of TheraSphere ® in combination with standard chemotherapy [NCT01483027].

### The Role of SIRT in Chemorefractory Patients

In patients with chemorefractory liver metastases, a randomized phase III study showed that SIRT prolonged the time to tumor progression in the liver, and time to overall progression when compared to chemotherapy 5-FU alone [34]. A systematic review in 2014, comprising 20 studies with 979 patients, reported that the rates of complete response, partial response, and stable disease were 0 (range 0 to 6), 31 (range 0 to 73), and 40.5 (range 17 to 78) percent, respectively. The median time to intrahepatic disease progression was 9 months, median time to disease progression overall 4.9 months, and median OS 12 months. Acute toxicity developed in a median 40.5%, mostly of grades 1 or 2. However, interpretation of these results is challenging as patient selection and treatment methods were varied [35].

Hepatic impairment after SIRT has been observed, presenting with liver failure or radioembolization-induced liver disease (REILD) manifesting as jaundice and ascites developing 1 to 2 months after treatment, in the absence of cancer progression or biliary obstruction. Potential longer term complications include liver fibrosis and portal hypertension. In a prospective study reviewing hepatotoxicity in 45 patients without chronic liver disease who underwent SIRT in 2003–2006, 20% developed REILD with the two of the most severe cases showing histological changes of veno-occlusive disease [36]. Risk factors for REILD include underlying cirrhosis or exposure to systemic chemotherapy within 2 months prior to treatment [37].

NCCN and ESMO guidelines [22, 38••] have included SIRT as a treatment option for liver-dominant, chemotherapy-resistant colorectal disease. The precise role of SIRT in earlier lines of treatment demands further evaluation.

## Transarterial Chemoembolization

Transarterial chemoembolization (TACE) also capitalizes on the differential blood supply to liver metastases and normal liver parenchyma; it causes occlusion of selected hepatic artery branches and allows maximum exposure of liver-directed chemotherapy to the ischemic environment formed within the target metastases.

A retrospective study evaluated the efficacy of repeated TACE performed in 560 patients with unresectable liver metastases. Embolization was performed with lipiodol and starch microspheres. Depending on prior systemic treatment received, the chemotherapy agent utilized for TACE differed—including mitomycin C alone (43.1%), mitomycin C in combination with gemcitabine (27.1%), or with irinotecan (15.6%), or with both irinotecan and cisplatin (15.6%). Partial response rate and stable disease rate were 16.7% and 48.2% respectively, while the median OS was 14.3 months [39].

A newer approach, which is generally well tolerated, employs drug eluting beads [40] and allows better controlled drug delivery to liver tumors. A phase III trial randomizing 74 previously treated patients with liver-confined colorectal metastases to TACE with irinotecan-loaded drug-eluting beads (DEBIRI) or systemic FOLFIRI [41] reported an OS advantage with DEBIRI of 22 months vs 15 months (*p* = 0.031). A 2013 meta-analysis of five observational studies and this randomized controlled trial evaluating DEBIRI and FOLFIRI concluded that DEBIRI was safe and effective, but further prospective clinical trials are needed [42]. A more recent study assigned 60 patients to FOLFOX and bevacizumab with or without DEBIRI, showing a significant improvement in response rates (78% vs 54%, *p* = 0.02) at 2 months, which was the primary endpoint [43]. At present, the Cochrane systematic review does not recommend TACE as a standard therapy outside of randomized clinical trials [44].

## Ablation

Thermal ablative techniques include radiofrequency ablation (RFA), microwave ablation (MWA), interstitial laser ablation, and cryotherapy. Cryoablation was one of the earliest ablative techniques used. It involves introducing liquid nitrogen or argon gas under ultrasound guidance to the tumor to disrupt tissue by freezing [45]. It has fallen out of favor due to significant toxicity to normal surrounding liver parenchyma and high recurrence rates [46] as well as higher bleeding risks.

### Radiofrequency Ablation

The most commonly employed ablative technique is RFA. It uses a high-frequency alternating electric current (radiofrequency 300–500 kHz) to cause frictional heating around the electrodes, destroying surrounding tissue. It can be performed percutaneously, laparoscopically, or via open surgery with low mortality and morbidity. In a systematic review of 36 studies, the major complication rates ranged from 0 to 33% [47]. RFA is most effective when applied to a solitary lesion or a few smaller lesions less than 3 cm in diameter as effective treatment requires necrosis of the targeted tumor together with a rim of normal liver tissue [48–50]. Placement near large blood vessels may result in the “heat sink effect” as blood flow dissipates heat away from the target tumor [51, 52]. Consideration of laparoscopic or open RFA should be made when treating lesions in the liver dome or along the inferior liver edge to reduce risks of diaphragmatic injury [53] and intestinal perforation.

### The Role of RFA in Resectable Disease

Retrospective studies have reported that RFA alone is inferior to resection in terms of local recurrence rates and OS in patients with oligo- liver or lung metastases [54, 55], especially in the absence of extrahepatic disease. However, there are no randomized controlled trials examining this issue directly. It is difficult to interpret retrospective analyses reliably given the varied patient characteristics and treatment-related factors across the different trials [56]. The 2010 ASCO clinical evidence review concluded, based on these heterogeneous retrospective studies and a limited number of prospective trials, that there is insufficient evidence to support the use of RFA over surgical resection in patients with potentially resectable colorectal cancer liver metastases, and that there is a compelling need for more research in this area [57]. Similar conclusions were reached in a 2012 Cochrane Database systemic review [58].

### The Role of RFA as an Adjunct

RFA as an adjunct has been shown to expand the group of patients eligible for resection with curative intent. A prospective phase II trial looking at 52 patients with unresectable liver metastases treated with RFA either with or without resection achieved a complete hepatic response in 75% of patients and a 43% 5-year OS [59]. In a large single institution retrospective analysis evaluating combined ablation and resection versus resection alone in patients with bilobar liver metastases [60], the combined approach was associated with improved perioperative outcome with less blood loss and shorter recovery time. The 5-year OS was similar (56% and 49%, *p* = 0.16) although it is noted that the combined treatment group had significantly poorer prognostic risk score. The ESMO guidelines also recognize the role of RFA in addition to surgery with the goal of eradicating all visible metastatic sites [38••].

### The Role of RFA in Addition to Systemic Chemotherapy for Unresectable Disease

The EORTC CLOCC trial [61], a phase II study that was originally designed to be phase III but met slow accrual, randomized 119 patients with unresectable liver-confined colorectal cancer metastases to systemic chemotherapy with or without RFA (± resection) in the first-line setting. RFA was the only local treatment in about half (53%) the patients, with the remainder receiving combined RFA and resection. PFS was significantly longer for patients who received RFA (± resection) at 16.8 months versus 9.9 months for patients who received chemotherapy only but there was no significant OS difference. However, the 10-year update demonstrated median OS of 45.6 months (95% CI 30.3–67.8) versus 40.5 months (95% CI 27.5–47.7), *p* = 0.01, in favor of RFA with systemic chemotherapy [62••]. While this may suggest potential long-term benefit of RFA to chemotherapy for patient with unresectable colorectal cancer liver metastases, further elucidation is needed.

### Microwave Ablation

MWA uses electromagnetic waves in the microwave energy frequency (300 to 300 GHz) to produce heat directly from the tissues in a wider and more homogeneous area. The shorter wavelength compared to RFA allows for more rapid heating and less loss of energy across different densities of tissues, with less susceptibility to the “heat sink effect” [63]. A large Chinese retrospective review of 1136 patients, of which majority had primary hepatocellular carcinoma while 86 had metastatic colorectal cancer, concluded that MWA is a well-tolerated technique [64]. In this study, major complications occurred in 2.6% of patients and included liver abscess and empyema formation, perforation of the colon, bile duct injury, tumor seeding, pleural effusions, hemorrhage requiring arterial embolization, and skin burns.

A retrospective matched cohort analysis at MSKCC of 134 patients comparing MWA to RFA showed that ablation site recurrences were lower in the MWA group (6% vs. 20%, *p* < 0.01) but there was no significant difference in OS [65]. There are presently no prospective randomized trials comparing these two techniques. In a randomized trial, 30 patients with potentially resectable colorectal cancer liver metastases were assigned to laparotomy with ultrasound-guided MWA or surgical resection [66]. Both groups had similar survival with a median survival of 27 and 25 months respectively.

MWA is not a readily accessible modality of treatment partly due to cost and local vendor availability. This technique needs to be studied further so that guidelines can be instituted and applied in more institutions across the globe.

### Laser-Induced Interstitial Thermotherapy

Laser-induced interstitial thermotherapy (LITT) directs laser radiation through thin optical fibers that are introduced into a targeted area by image guidance and destroys tissue by thermal effect. A large German series involving 603 colorectal cancer patients with five or fewer liver metastases, smaller than 5 cm, who underwent MRI-guided laser thermotherapy for colorectal cancer liver metastases demonstrated less than 5% local recurrence at 6 months and a 5-year OS of 37% [67]. However, LITT is not widely available, and use presently remains limited to a few institutions.

### Irreversible Electroporation

Irreversible electroporation (IRE) is a relatively new technique still in the early stages of development. Its safety for ablating tumors in humans was first reported in a single-center nonrandomized cohort study in 2011 [68]. IRE uses a high-voltage electric current to create nanopores in the cell membrane, leading to loss of homeostasis and resulting in apoptosis [69]. As IRE is non-thermal, it may be a good modality to treat tumors in close proximity to major vessels or biliary ducts [70]. At present, costs are prohibitive and guidelines for application are still lacking.

## Combination with Immunotherapy

Radiation therapy and thermal ablative therapies are considered immunogenic and in this era of immunotherapy, multiple studies are in progress to test the synergistic effect of combination treatment, with promising preclinical data and case reports [71]. A pilot study evaluating AMP-224, a PD-1 inhibitor, in combination with SBRT for patients with colorectal cancer liver metastases demonstrated safety although no responses were observed [72]. A phase II trial at MSKCC evaluating either radiotherapy or radiofrequency ablation combined with pembrolizumab (NCT02437071) reported interim results at the ASCO 2016 Annual Meeting with one patient in the radiotherapy cohort achieving partial response while there were no responses in the ablation cohort. Results of ongoing trials are highly anticipated.

## Conclusion

Clinical outcomes for patients with colorectal cancer liver metastases have improved over the last two decades, as more patients undergo potentially curative resection, and as our armamentarium of systemic treatment and liver-directed therapies continue to expand in both palliative and curative settings.

Resection of hepatic metastasis remains the gold standard to remove macroscopic disease in colorectal cancer patients with liver-only metastases. However, not all patients present with clearly resectable hepatic disease, and there are limitations to the extent of response systemic therapy can achieve presently. As such, several techniques have been developed as adjuncts to improve resectability in patients who are unable to meet the resectability criteria. They may also be employed in the adjuvant setting to potentially reduce local recurrence rates, as well as in the palliative setting to treat clinically critical disease with the aim to improve overall survival.

Patients with limited liver metastases but not meeting the resectability criteria should consider tumor ablation with radiofrequency ablation keeping in mind that it is most effective when applied to patients with a solitary lesion or a few smaller lesions each less than 3 cm in diameter located away from large blood vessels. While SIRT is not recommended in the first-line setting in addition to oxaliplatin-containing chemotherapy, it can be considered for patients who have failed multiple lines of systemic chemotherapy. Adjuvant hepatic artery infusion is an option with the consensus that it should be considered only at institutions with experience in the procedure given potential surgical complications and drug-related toxicity. Some of the other therapies discussed above such as microwave ablation are also relatively novel and have yet been widely applied partly due to technical constraints. All therapies should be considered in centers with adequate expertise in the specific techniques, after multidisciplinary team discussions, taking into account the extent of disease, underlying liver function, treatment goals, and the toxicities of treatment.

Ongoing research is expected to explore and validate the role of these evolving therapeutic options, and prospective randomized trials are essential to determine how best to sequence and when to employ these therapies.
